# CCL2/MCP-I Genotype-Phenotype Relationship in Latent Tuberculosis Infection

**DOI:** 10.1371/journal.pone.0025803

**Published:** 2011-10-04

**Authors:** Rabia Hussain, Ambreen Ansari, Najeeha Talat, Zahra Hasan, Ghaffar Dawood

**Affiliations:** 1 Department of Pathology and Microbiology, Aga Khan University, Karachi, Pakistan; 2 Masoomeen Trust Hospital, Karachi, Pakistan; University of Delhi, India

## Abstract

Among the known biomarkers, chemokines, secreted by activated macrophages and T cells, attract groups of immune cells to the site of infection and may determine the clinical outcome. Association studies of CCL-2/MCP-1 -2518 A/G functional SNP linked to high and low phenotypes with tuberculosis disease susceptibility have shown conflicting results in tuberculosis. Some of these differences could be due the variability of latent infection and recent exposure in the control groups. We have therefore carried out a detailed analysis of CCL-2 genotype SNP -2518 (A/G transition) with plasma CCL-2 levels and related these levels to tuberculin skin test positivity in asymptomatic community controls with no known exposure to tuberculosis and in recently exposed household contacts of pulmonary tuberculosis patients. TST positivity was linked to higher concentrations of plasma CCL2 (Mann Whitney U test; p = 0.004) and was more marked when the G allele was present in TST+ asymptomatic controls (A/G; p = 0.01). Recent exposure also had a significant effect on CCL-2 levels and was linked to the G allele (p = 0.007). Therefore association studies for susceptibility or protection from disease should take into consideration the PPD status as well as recent exposure of the controls group used for comparison. Our results also suggest a role for CCL-2 in maintaining the integrity of granuloma in asymptomatic individuals with latent infection in high TB burden settings. Therefore additional studies into the role of CCL-2 in disease reactivation and progression are warranted.

## Introduction

One third of the world' population is latently infected with *M. tuberculosis* and forms the largest pool contributing to new cases. Among the 20 high TB burden countries, Pakistan ranks 8^th^ despite >95% coverage with BCG vaccination at birth [WHO/UNICEF, 2009]. In Pakistan, latent infection as assessed by tuberculin skin tests (TST) in the community has been reported to be as high as 40–50% [1]and even higher(70–80%) in the exposed household contacts [2]. The highest number of TB cases is contributed by this pool of latently infected individuals. It is therefore important to understand the molecular determinants associated with latent infections and active disease in tuberculosis in this high TB endemic setting.

TST was introduced a century ago and is the oldest test for detection of cell mediated immunity in tuberculosis and is therefore considered to be a marker of latent infection in asymptomatic individuals. TST positivity can be affected by conditions which compromise the immune system in the host such as poor nutrition, advanced age and infections such as HIV and diabetes. Additional surrogate immune markers associated with latent infections or individuals at risk of developing latent infection would be of tremendous help to National TB programs.

Among the immune markers, chemokines play an important role in the initial stages of infection by recruiting leukocytes to the infected or inflamed sites in the peripheral tissues [3]. Chemokines participate in the growth, differentiation and activation of leukocytes and stimulate various effector functions such as chemotaxis, integrin activation, superoxide radical production and granule enzyme release. Chemokines, therefore, serve as critical components of basal leukocyte trafficking essential for immune system architecture, development and immune surveillance [3].

One such C-C chemokine CCL-2, is produced by activated T cells and macrophages and exclusively recruits monocytes and T cells to the site of infection in mycobacterial diseases. CCL-2 is the most potent activator of macrophages where it derives its earlier name of macrophage chemoattractant protein-1 (MCP-1) [4]. Since mycobacteria reside and multiply within the macrophages, levels of macrophage recruiting chemokines may play a crucial role in effective recruitment of and activation of appropriate effector T cells at the site of infection. CCL-2 predominantly recruits memory T cells to the site of infection and therefore may play an important role in maintaining the architecture of granuloma in latent infection.

A single nucleotide polymorphism (-2518 A/G) in the CCL-2/MCP-1 gene, controls the level of CCL-2 protein [5]. Cells with the homozygous AA genotype secrete significantly less CCL-2 than do cells with either AG or GG genotype [5], [6]. CCL-2 (-*2518G→A*) SNP has shown variable association of *G^hi^* allele with either susceptibility to TB [6], [7] or no association with TB [8]–[10], (see Table S1). Some of these differences could be due to differences in TST status of the controls used for comparison with TB groups which included blood donors [8], community controls with unknown TST status [6], [11]–[13] [6] or TST positive controls [14]. There is variability in the frequency of genotypes [11] as well as in the prevalence of latent infection in populations and therefore it is difficult to compare controls between studies. Only one report studied the association of plasma levels with CCL-2 -2518 SNPs and found no difference in the CCL-2 plasma levels in healthy tuberculin reactors and non reactors. [6]. However, genotype analysis in relation to CCL-2 in tuberculin reactors and non reactors was not done.

In the current study we have carried out a detailed analysis of CCL-2 genotype SNP -2518 (A/G transition) with plasma CCL-2 levels in asymptomatic controls with no known exposure to tuberculosis, and in recently exposed household contacts of pulmonary tuberculosis patients and related these levels to tuberculin skin test positivity. Plasma CCL-2 levels showed no relationship with CCL-2 -2518 SNP in the asymptomatic controls. However, TST positivity was associated with higher concentrations of plasma CCL-2 and was more marked when the G allele was present in both household contacts and the community control groups. Therefore CCL-2 genotype and phenotype may play an important role in both establishment of latency and maintenance of granuloma.

## Materials and Methods

### Ethical statement

A name de-linked DNA bank and paired plasma samples were available on exposed household contacts (HC) and community based healthy controls (EC) in the department of Pathology and Microbiology through previously funded projects. Ethical approval by The Aga Khan University Ethical Review Committee was obtained for the project (1451-Path-ERC-2010).

### Study groups

This study group consisted of BCG vaccinated, asymptomatic healthy controls (N = 196). Detailed information was available on each household contact with respect to previous exposure, anti-tuberculous treatment (ATT), immune modifying treatment and co-morbidities which may affect immune responses such as diabetes, HIV etc. Household contacts (N = 110) of tuberculosis patients were living with a pulmonary tuberculosis patients pretreatment for a minimum of 3 months. Endemic community controls (EC = 86) had no recent history of exposure or previous history of tuberculosis treatment.

### Tuberculin skin tests

Tuberculin skin tests (TST) were carried out by injecting 5 TU intra-cutaneously in the volar surface of the arm (Tubersol ^tm^, purchased from Sanofi Pasteur Limited, Ontario Canada). A single tester administered and read the indurations after 48 hours using a caliper. A diameter ≥10 mm was considered positive [15]. Written consent was obtained from each participant.

### Collection of plasma

Five ml of blood was collected by venipuncture from each donor and mixed with sodium heparin (20 U/ml; Leo pharmaceutical, Ballerup, Denmark) in 15 ml of plastic centrifuge tubes. Red blood cells were allowed to settle and plasma from whole blood was collected and stored as small aliquots at −80°C until use.

### Assessment of CCL-2 levels in plasma

Chemokine levels in plasma were estimated using Flow Cytometric Bead Array (CBA) from BD Biosciences Ca, USA. This equipment allows detection of several cytokines or chemokines, simultaneously in small volumes of samples and is kit based. The principle of CBA is the use of beads with discrete fluorescence intensities. Each bead with a single fluorescent intensity is coated with a different monoclonal capture antibody and serves to capture the soluble analyte present in plasma, serum, stimulated culture supernatants, cell lysate or other body fluids. A reporter antibody that is attached to fluorescence molecule of phycoerythrin [PE] is used to quantify the captured analyte of interest. Kits are available for a combination of chemokines and cytokines. Chemokine Kit (Human chemokine kit) was purchased from BD biosciences. This kit detects 5 related chemokines (CXCL-8/IL-8; CCL5/RANTES; CXCL-9/MIG; **CCL-2**; CXCL10/IP10). Plasma was diluted 1:5 and in-filter plate assay was used. The sensitivity was in the range of 2.5–5.0 pg/ml as given by the manufacturer.

### Molecular analysis

#### DNA Extraction

Five ml blood was collected in ACD tubes (VWR Scientific, West Chester, PA, USA). Genomic DNA was extracted from whole blood as described in detail previously [16]. Promega Wizard Genomic DNA Purification Kit (Promega Corporation Madison, WI, USA) was used according to the manufacturer's instructions. DNA was quantified by spectrophotometer and stored at −35°C until further analyses.

#### Detection of CCL-2 SNPS

CCL-2 genotype SNPs (-2518) analyses were carried out using conventional PCR followed by sequencing of the PCR products. The primers were designed using web based software “BatchPrimer3”. Primers were purchased from MWG-Biotech AG, (Ebersberg, Germany) (CCL-2 Forward: TCT GGG AAC TTC CAA AGC TG & CCL-2 Reverse: GCC ACA ATC CAG AGA AGG AG). After completion of 25 PCR cycles, 5 µl of each PCR product was mixed with 1 µl of 6× bromophenol blue loading dye. Products with dye were loaded on 2% agarose gel prepared in Tris-acetate EDTA (TAE) buffer with 10 mg/ml ethidium bromide. 100 base pair molecular marker was also loaded in one of the wells for reference. These products were then run for 45 minutes at 120 volts in electrophoretic tank containing TAE buffer. Product bands were visualized on a UV-transilluminator and pictures were taken of the visible bands (**Figure S1**). PCR products (25–30 µl) were sent to Macrogen (Macrogen Inc, Seoul, Korea) for sequencing. Sequencing results were analyzed by multiple alignments of the sequences using software “Clustal W version 1.83”. CCL2 genotypes (-2518) were calculated by referring chromatograms of the sequencing results provided by Macrogen.

### Statistical analysis

Computer software SPSS version16.0 and Epi Info 2000 applications were used to carry out statistical analyses. Kruskall –Wallis was applied to test for differences among related groups. Mann-Whitney U test was applied when N was <20 in any group and student t test when the N was >20 to determine significant differences between groups. For categorical data, Chi square test was applied. Allelic Frequencies were compared between groups by Pearson chi-squared tests or Fisher's exact tests when analyzing allelic frequencies lower than five to determine statistical significance differences between groups. Odds ratios (OR) with respective confidence intervals (95% CI) for disease susceptibly were also calculated. Linear-by-linear test were used to determine the significance (corrected p values) of genotypes between healthy controls and disease groups. Values of *p*<0.05 were considered significant for both Pearson and linear-by-linear χ^2^ test.

## Results

### Characteristics of the study groups and subgroups

Asymptomatic donors with no history of tuberculosis treatment (Tuberculosis Not Affected individuals; TBNA = 196) and where CCL2 genotype SNPs analysis was available, were included in the study. TBNA was further stratified into subgroups (**Table 1**) of: 1) BCG vaccinated endemic controls (EC = 86) with no history of recent exposure to active tuberculosis patients and 2) recently exposed household contacts (HC = 110) living with an index case of open pulmonary tuberculosis for at least 3 months [17] and were asymptomatic at the time of intake. There was no difference in age or gender among the two groups (chi test, P>0.1) (**Table 1**). The availability of TST status in EC and HC is also given in Table 1. Positive TST (≥10 mm indurations) in the HC group (75%) was 1.5 -fold higher compared to the EC group (46%). We first addressed the issue if there was an association of CCL-2 -2518 SNP with CCL-2 plasma levels in the asymptomatic group. Paired CCL2 genotype and phenotype was available in a subset of TBNA (N = 142).

**Table 1:** Characteristics of the study group.

Group ID	N	Age ± SD	Sex ratio M/F (ratio)	*TST status N positive/N tested (% positive)
TBNA	196	27.91±12.58	90/106 (0.85)	117/184 (61%)
EC	86	29.12±7.63	33/53 (0.62)	34/74 (46%)
HC	110	26.96±15.35	57/53 (1.08)	83/110 (75%)

Note: TBNA =  TB not affected:

TBNA Subsets = EC = Community Controls, HC exposed household contacts.

*Cut off for TST positivity ≥10 mm of indurations.

### No association of Plasma CCL-2 levels with CCL-2 genotype in TBNA

**Figure 1:**
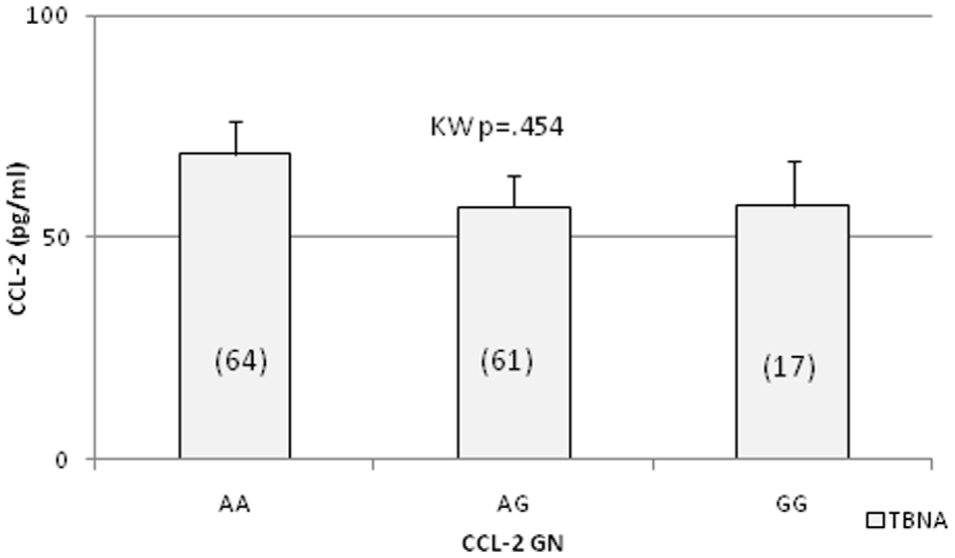
CCL-2 genotype-phenotype relationship in healthy asymptomatic control groups. PCR and sequencing analyses was carried out to determine the CCL-2 (SNP -2815) (see Figure S1) and BD FACS array was carried out to determine CCL-2 protein in plasma samples. The number in each genotype is indicated in the brackets. Kruskall-Wallis (KW) analysis was applied to determine significant differences across groups.

**Figure 1** shows the effect of CCL-2 -2518 SNPS on plasma CCL-2 levels in asymptomatic group (TBNA = 142). CCL-2 plasma levels were similar in donors irrespective of the genotypes (Kruskall-Wallis p = 0.454).

### Higher plasma CCL-2 levels are significantly associated with latency

Plasma CCL2 levels were available in 157 TBNA donors. We analyzed CCL-2 plasma levels in groups stratified on the basis of 1) remote (EC = 65) or recent (HC = 92) infection and 2) TST status in these groups (Table 2). TBNA showed the most significant difference in the mean plasma CCL-2 levels when groups were stratified on the basis of TST status (p = 0.004). The effect of recent infection on CCL2 plasma levels compared to remote infection was only marginal (p = 0.052) and when these two groups were restricted by TST positivity there was no significant difference in CCL-2 levels in the two groups. These results suggest that that elevated CCL-2 plasma levels in the HC group may associated with the higher rate of latent infection (HC 75% Vs EC 46% TST+) rather than recent exposure.

**Table 2:** CCL-2 levels in plasma in relation to infection and disease in tuberculosis.

Groups (N)	N	Mean (pg/ml)	±SE	p =
TBNA	157	59.8	4.5	
TBNA (+)	91	71.6	6.2	
TBNA (−)	49	47.4	6.3	0.0040
EC	65	45	9.2	
HC	92	70.1	3.7	0.052
EC (+)	23	67	21.1	
HC (+)	68	73.2	4.4	0.3

Note: TBNA = TB not affected:

TBNA Subsets = EC = Community Controls; HC exposed household contacts.

(+) = TST positive (> = 10 mm indurations); (−) = TST negative (<10 mm indurations).

Significance of differences was assessed by applying student t tests (1 tail, type 3).

A p value of <0.05 was considered significant.

### CCL-2 -2518 GG genotype is overrepresented in the household contacts compared to community controls

We next compared CCL-2 (-2518 SNP) genotypes within each of these subgroups (**Table 3**). Genotypes were in Hardy-Weinberg Equilibrium (HWE) in all groups. Although the highest difference in plasma CCL-2 levels was observed between TST+ and TST− TBNA (MWU p = 0.004), there was no difference in the genotype or allele frequency between these two groups. Surprisingly, the only groups showing significant genotype difference were the HC group compared to the EC group (χ2 = 8.98; p = 0.003) with an overrepresentation of G allele in the HC group, although the plasma CCL-2 levels were only marginally different (p = 0.052; **Table 2**) in these two groups. Since the HC group had much higher rate of latent infection compared to EC, these results suggest that the household contacts were at a higher risk of developing latent infection due to the presence of CCL-2 -2518 GG genotype. We therefore also analyzed the linkage of plasma CCL-2 with different genotypes in different groups in relation to TST status.

**Table 3:** CCL-2 (-2518 G/A) genotype and allele analysis.

CCL-2 – 2518	TBNA (+)	TBNA (−)	EC	HC	EC(+)	HC(+)
SNP Genotype	*f*	*f*	*f*	*f*	*f*	*f*
N	117	67	86	110	34	82
AA	41.88	49.30	45.35	43.64	44.12	41.46
AG	43.59	44.80	51.16	39.03	50	40.2
GG	14.53	15.97	3.49	17.27	5.88	18.29
X^2^		2.39		***8.98***		2.89
Corrected		Vs TBNA+		**Vs EC**		Vs EC+
p =		.122		***0.003***		0.089
allele						
A	63.675	71.6	70.93	63.18	69.12	62.59
G	36.325	28.4	29.07	36.82	30.88	38.41
X^2^		2.42		2.6		1.18
p =		.21		.107		.277
OR		.69		.7		.72
95% CI		0.44–1.1		0.46–1.08		0.39–1.31
HWE p =	.876	.615	.957	.515	.876	.615

Note: TBNA = TB not affected:

TBNA Subsets = EC = Community Controls, HC exposed household contacts.

TST positive = (+); TST negative = (−). TST positivity cut off = ≥10 mm indurations.

HWE = Hardy Weinberg Equilibrium. P<0.05 is considered significant.

### Genotype-phenotype relationship of CCL-2 -2518 SNPS in TBNA stratified by TST status

**Figure 2:**
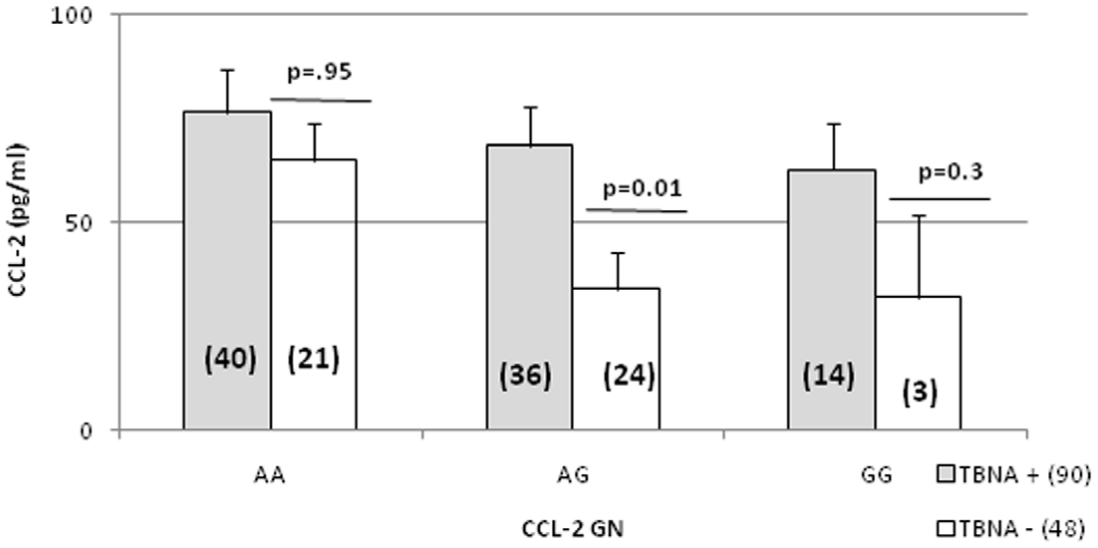
CCL-2 genotype-phenotype relationship in the presence (TST+) or absence (TST-) of latency in asymptomatic healthy controls. TST+ (cut off > = 10 mm diameter indurations). The number in each panel is indicated in the boxes. Significant differences were determined by Mann Whitney U analysis between different genotytpes. A p value <0.05 was considered significant. Genotypes were unavailable on 2 donors and therefore not included. All other parameters are same as in table 2 and 3.

The effect of CCL-2 genotypes on CCL-2 plasma levels was first examined in TBNA according to their TST status (**Figure 2**). TST+ donors with AA genotype showed comparable responses in both TST+ and TST− groups. However, there was a significantly higher level of plasma CCL-2 in the TST+ donors when G allele was present. Due to a low number of donors with GG genotype in TST-, there was insufficient numbers to carry out meaningful statistical analysis but there was a clear overrepresentation of GG genotype in TST+TBNA compared to TST-TBNA indicating that donors with G allele may be at a higher risk of developing latent infection and that this may be dose dependent.

### Genotype-phenotype relationship of CCL-2 -2518 SNPS in HC group compared to EC group

**Figure 3:**
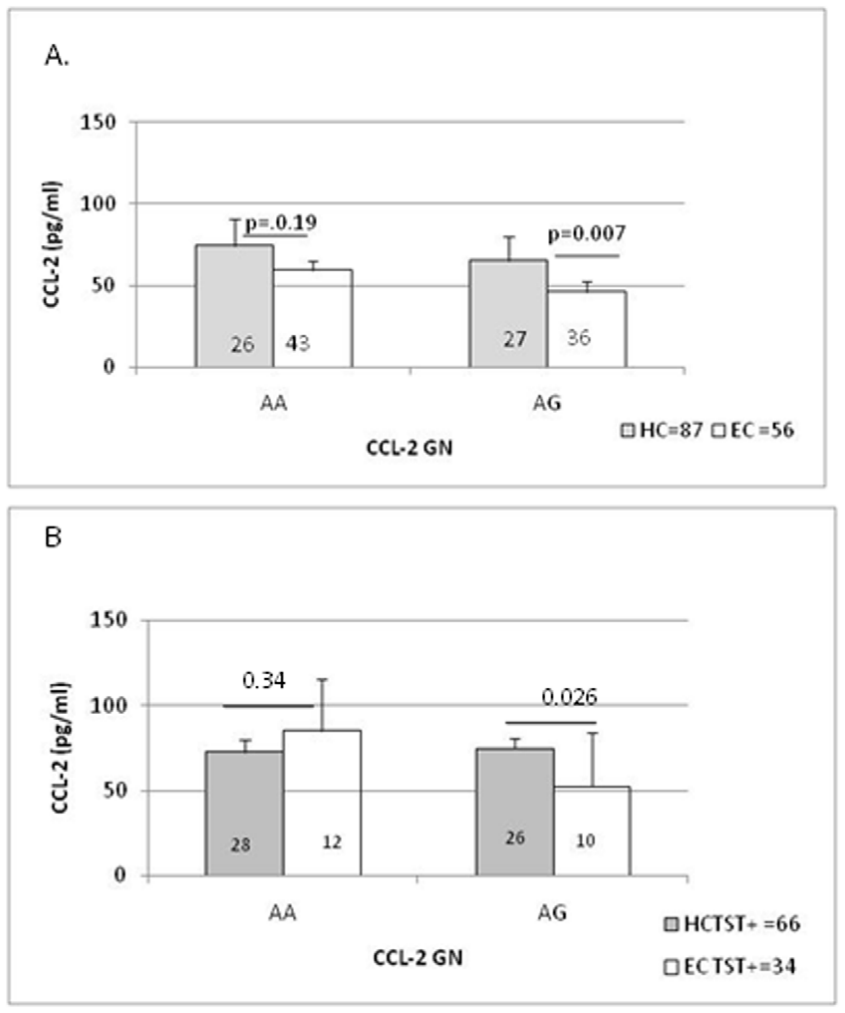
CCL-2 genotype-phenotype relationship in the presence (TST+) or absence (TST-) of latency in recently exposed household contacts (Panel A) and healthy community controls (Panel B). The number in each group is indicated in the boxes. Significant differences were determined by Mann Whitney U analysis between different genotytpes. A p value <0.05 was considered significant.

We next compared the effect of CCL-2 genotype in TBNA stratified either on the basis of exposure (EC = remote Vs HC = recent) (Figure 3 A) or TST status (Figure 3 B). We have given results for only AA and AG genotypes as there were insufficient numbers of donors with GG genotype in the EC group for meaningful statistical analysis. The differences in CCL-2 plasma levels in the HC and EC groups were much more marked when G allele was present (p = 0.007). This difference became less marked (p = 0.026) when the groups were restricted by TST positivity (Figure 3B). Again donors with AA genotype showed comparable responses irrespective of the TST status. These results therefore show that higher CCL-2 levels are present in latently infected individual and that the presence of G allele may further boost CCL-2 plasma levels in household contacts.

### Receiver Operator Curves (ROC) analysis between EC and HC groups

We next analyzed the discriminating power of plasma CCL-2 levels in community controls and household contacts. Receiver Operator Curves were generated for comparison between different groups and AUROC and p values are shown in **Table 4**. As expected the CCL-2 showed the best discrimination between EC− and HC+ groups (AUROC = 0.736; p = 0.0003) and slightly less between the EC− group and the unstratified HC group (AUROC = 0.694; p = 0.002). The least discrimination was observed when the comparison was restricted to TST+ in EC and HC group again indicating an association of CCL2 levels with TST positivity.

**Table 4:** Receiver Operator analysis of CCL2 (pg/ml) in Healthy Asymptomatic Community Controls in relation to Tuberculin Skin test Status (TST).

Groups	N	AUROC	p =	CI
EC− Vs HC +	27 Vs 80	.736	0.0003	0.605–.867
EC− VS HC	27 Vs 104	.694	0.002	.571–.817
EC+ Vs HC+	24 Vs 80	.658	0.019	.172–.512

EC = Community Controls; HC exposed household contacts. + = TST positive (> = 10 mm indurations);

− = TST negative (<10 mm indurations).

## Discussion

There is a high rate of latency as assessed by TST positivity in TB endemic setting which is attributed to BCG vaccination, exposure to environmental mycobacteria or high rate of transmission within the community. The host immune factors which influence this high rate of latent infection in an endemic setting are as yet incompletely understood.

Tuberculin Skin Tests (TST) was 1.5-fold higher in the HC group compared to the EC group. The higher rate of TST positivity in household contacts [17] could either be due to recent exposure or a higher genetic susceptibility for latent infection as all household contacts were blood relatives of tuberculosis patients. In TB endemic countries the high rate of TST+ positivity particularly in the community controls has been attributed to factors other than recent exposure such as BCG vaccination or exposure to environmental mycobacteria which may boost T central memory cells in BCG vaccine's [18]. Similarly interferon gamma release assays (IGRA) which test for Th1 subset activation have failed to discriminate latent infection from recent exposure and have very little added value in an endemic setting [19]. Surrogate biomarkers for latent infection have therefore been an area of intense investigation which include an array of stimulated cytokines [20] [2]. Less attention has been paid to chemokines which are considered to be derived from the innate arm and hence less specific. However, there has been increasing interest in chemokines as markers of infection and disease [21]. Among the chemokines, CXCL10/IP10, a chemokine induced by IFN- γ has shown promising results as marker of tuberculosis disease in children [22]. We have also previously reported that among the several endogenously and exogenously activated chemokines [23]–[26] CCL2 is elevated in the more severe form of tuberculosis [23]. Kinetic patterns of chemokine and cytokine profile in *M. bovis* BCG induced chemokines from human peripheral blood mononuclear cells show that CCL2 is released early post stimulation while CXCL10/IP10 is released later and this parallels TNF-α which is released early while IFN-γ which is released later indicating that the expression of both chemokines and cytokines is regulated [21]. Since CCL2 is released early we have focused on the role of CCL2 in mycobacterial latent infection which is the earliest consequence of susceptibility to infection.

In the current study, we show that the CCL-2 -2518 G^hi^ allele was associated with an increased risk of latency, as this genotype was overrepresented in the HC group compared to the community controls and may contribute to increased genetic susceptibility in the household contacts.

CCL-2 is a member of the small inducible gene (SIG) family which is induced under conditions of oxidative stress, by cytokines and or growth factors. CCL-2 chemokine is believed a play an important role in both establishment and maintenance of granuloma in latent infection as chemokine are pivotal in recruitment of leukocytes to the site of infection [4]. A polymorphism in the CCL-2 gene (-2518 A/T), differentially controls the level of CCL-2 protein [5]. Several studies have associated the G allele with increased expression of CCL-2 in plasma [27] [28] [6] [29], other studies have reached the opposite conclusion [30] [31] and some studies have failed to find a significant difference in the plasma CCL-2 levels in individuals harboring either allele [30]. In our study while plasma CCL-2 levels showed no relationship with CCL-2 genotype in the asymptomatic controls (N = 196; Kruskall-Wallis p = .454), a significant difference in CCL-2 plasma levels was detected between TST+ and TST− donors in the asymptomatic group which was most marked when the G allele was present. One likely explanation may be the expression of specific Prep/Pbx proteins complexes which regulate CCL-2 expression in different cells/tissues during different infectious states [32].

The biological role of CCL-2 in tuberculosis has been addressed in a previous study [6] where the investigators have shown that stimulation of healthy monocytes from -2518GG with *Mycobacterium tuberculosis* antigens yielded higher CCL-2 levels and lower IL-12p40 compared with healthy -2518AA genotype. Therefore, the investigators concluded that -2518 GG homozygote produce higher concentrations of CCL-2 and suppress IL-12p40 production responsible for deriving the IFN-γ response. IFN- γ is a key macrophage activating cytokine and has been shown to be a central molecule in protection against mycobacteria.

Furthermore, *In vivo* association studies of CCL-2 SNPs with susceptibility to tuberculosis in different ethnic populations also support the above observations. Flores -Villanueva et al found an increased risk of developing tuberculosis in Mexicans and Koreans heterozygous or homozygous for CCL-2 -2518G (rs1024611) compared with those homozygous for -2518A allele [6]. ELISA analysis showed that TB patients homozygous for -2518G had the highest plasma levels of CCL-2 and the lowest plasma levels of IL12p40, and these values were negatively correlated. Stimulation of monocytes from healthy -2518G homozygotes with *M. tuberculosis* antigens also yielded higher CCl-2 and lower IL12p40 compared with healthy -2518A homozygotes. Addition of anti-CCL-2 antibodies increased IL12 production by -2518G homozygotes, whereas addition of CCL-2 reduced IL12 production by -2518A homozygotes. The investigators [6] concluded that those with the CCL-2 -2518 GG genotype produce high concentration of CCL-2, which inhibits IL12p40 production in response to *M. tuberculosis* and increases the likelihood of TB infection progressing to active disease. We have reported that IFN-γ -874 T ^hi^ allele was associated with less severe pulmonary disease but not with severe pulmonary or extra pulmonary tuberculosis [33] while CCL-2 responses show the opposite trend and are higher in severe disease compared to less severe disease [23]. Increased levels of CCL2 in TST+ donors in the current study are consistent with our previous report of higher CCL2 in TST+ EC compared to TST− EC [25] and lower IFN-γ levels in TST+ HC compared to TST+ EC [34].

CCL-2 acts predominantly on activated/memory stages of T cells [35]. CCL-2 is the only chemokines which exerts chemotactic effect on both Th1 and Th2 cells [36]. Furthermore CCL-2 is able to polarize Th0 phenotype to Th2 phenotype by up regulating IL-4 transcription thus indicating that CCL-2 may be part of the Th2 circuit [37]. The major purpose of granuloma is to create a focus of inflammatory cells that prevents inflammation from occurring systemically and maintaining a pro-inflammatory/anti-inflammatory balance within the granuloma to reduce pathology. Excessive levels of CCL-2 may however lead to increase risk of infection and disease progression by virtue of its ability to activate the Th2 circuit. In TB endemic regions concomitant infections with helminths can also skew the immune response towards Th2 and may enhance CCL2 responses which forms part of the Th2 loop. This study is set in a peri-urban area of Karachi, where the incidence of helminth infections is relatively low and as such should have little effect in skewing Th1/Th2 response. We have not screened our healthy donors for common parasitic infections. Nevertheless, all healthy donors were derived from the same socio-economic strata and therefore, it is unlikely that the incidence of parasitic infections will be different in TST− and TST+ donors. Paradoxically our results indicate higher plasma CCL2 responses in TST+ (Th1 associated) donors rather than TST− donors.

In conclusion we have identified an association of elevated CCL-2 plasma levels with latent tuberculosis and a further effect of GG genotype in increasing the risk of latent infection. This association was irrespective of remote or recent infection. This is not surprising as CCL-2 plays an important role in recruitment of T memory cells to the site infection which are long lived in a BCG vaccinated population [38]. Variable associations in different ethnic population may be due to both variability in the prevalence of latent infection, as well as variability of frequencies of the different CCL-2 genotypes.

## Supporting Information

Figure S1 **CCL2 PCR optimization for Sequencing Reactions.**
(TIF)

Table S1 **Frequency of CCL2 SNPS in reported in different populations.**
(DOCX)

## References

[1] Rathi SK, Akhtar S, Rahbar MH, Azam SI (2002). Prevalence and risk factors associated with tuberculin skin test positivity among household contacts of smear-positive pulnionary tuberculosis cases in Umerkot, Pakistan.. Int J Tuberc Lung Dis.

[2] Hussain R, Talat N, Shahid F, Dawood G (2009). Biomarker changes associated with Tuberculin Skin Test (TST) conversion: a two-year longitudinal follow-up study in exposed household contacts.. PLoS ONE.

[3] Gale LM, McColl SR (1999). Chemokines: extracellular messengers for all occasions?. Bioessays.

[4] Deshmane SL, Kremlev S, Amini S, Sawaya BE (2009). Monocyte chemoattractant protein-1 (MCP-1): an overview.. J Interferon Cytokine Res.

[5] Rovin BH, Lu L, Saxena R (1999). A novel polymorphism in the MCP-1 gene regulatory region that influences MCP-1 expression.. Biochem Biophys Res Commun.

[6] Flores-Villanueva PO, Ruiz-Morales JA, Song CH, Flores LM, Jo EK (2005). A functional promoter polymorphism in monocyte chemoattractant protein-1 is associated with increased susceptibility to pulmonary tuberculosis.. J Exp Med.

[7] Ben-Selma W, Harizi H, Boukadida J (2011). MCP-1 -2518 A/G functional polymorphism is associated with increased susceptibility to active pulmonary tuberculosis in Tunisian patients.. Mol Biol Rep.

[8] Chu SF, Tam CM, Wong HS, Kam KM, Lau YL (2007). Association between RANTES functional polymorphisms and tuberculosis in Hong Kong Chinese.. Genes Immun.

[9] Alagarasu K, Selvaraj P, Swaminathan S, Raghavan S, Narendran G (2009). CCR2, MCP-1, SDF-1a & DC-SIGN gene polymorphisms in HIV-1 infected patients with & without tuberculosis.. Indian J Med Res.

[10] Moller M, Nebel A, Valentonyte R, van Helden PD, Schreiber S (2009). Investigation of chromosome 17 candidate genes in susceptibility to TB in a South African population.. Tuberculosis (Edinb ).

[11] Larcombe LA, Orr PH, Lodge AM, Brown JS, Dembinski IJ (2008). Functional gene polymorphisms in canadian aboriginal populations with high rates of tuberculosis.. J Infect Dis.

[12] Thye T, Nejentsev S, Intemann CD, Browne EN, Chinbuah MA (2009). MCP-1 promoter variant -362C associated with protection from pulmonary tuberculosis in Ghana, West Africa.. Hum Mol Genet.

[13] Xu ZE, Xie YY, Chen JH, Xing LL, Zhang AH (2009). [Monocyte chemotactic protein-1 gene polymorphism and monocyte chemotactic protein-1 expression in Chongqing Han children with tuberculosis].. Zhonghua Er Ke Za Zhi.

[14] Buijtels PC, van de Sande WW, Parkinson S, Petit PL, van der Sande MA (2008). Polymorphism in CC-chemokine ligand 2 associated with tuberculosis in Zambia.. Int J Tuberc Lung Dis.

[15] Crofton J (1990). Crofton and Douglas Respiratory Diseases.. Clinical features of tuberculosis.

[16] Ansari A, Talat N, Jamil B, Hasan Z, Razzaki T (2009). Cytokine gene polymorphisms across tuberculosis clinical spectrum in Pakistani patients.. PLoS ONE.

[17] Talat N, Shahid F, Dawood G, Hussain R (2009). Dynamic changes in biomarker profiles associated with clinical and subclinical tuberculosis in a high transmission setting: a four-year follow-up study.. Scand J Immunol.

[18] Cardona PJ (2009). A dynamic reinfection hypothesis of latent tuberculosis infection.. Infection.

[19] Denkinger CM, Dheda K, Pai M (2011). Guidelines on interferon-gamma release assays for tuberculosis infection: concordance, discordance or confusion?. Clin Microbiol Infect.

[20] Menzies D, Pai M, Comstock G (2007). Meta-analysis: new tests for the diagnosis of latent tuberculosis infection: areas of uncertainty and recommendations for research.. Ann Intern Med.

[21] Mendez-Samperio P (2008). Expression and regulation of chemokines in mycoabcterial infection.. Journal of Infection.

[22] Liu M, Guo S, Hibbert JM, Jain V, Singh N (2011). CXCL10/IP-10 in infectious diseases pathogenesis and potential therapeutic implications.. Cytokine Growth Factor Rev.

[23] Hasan Z, Cliff JM, Dockrell HM, Jamil B, Irfan M (2009). CCL2 responses to Mycobacterium tuberculosis are associated with disease severity in tuberculosis.. PLoS ONE.

[24] Hasan Z, Jamil B, Ashraf M, Islam M, Yusuf MS (2009). ESAT6-induced IFNgamma and CXCL9 can differentiate severity of tuberculosis.. PLoS ONE.

[25] Hasan Z, Jamil B, Zaidi I, Zafar S, Khan AA (2006). Elevated Serum CCL2 concomitant with a reduced Mycobacterium-induced response leads to disease dissemination in leprosy.. Scand J Immunol.

[26] Hasan Z, Zaidi I, Jamil B, Khan MA, Kanji A (2005). Elevated ex vivo monocyte chemotactic protein-1 (CCL2) in pulmonary as compared with extra-pulmonary tuberculosis.. BMC Immunology.

[27] Fenoglio C, Galimberti D, Lovati C, Guidi I, Gatti A (2004). MCP-1 in Alzheimer's disease patients: A-2518G polymorphism and serum levels.. Neurobiol Aging.

[28] Gonzalez E, Rovin BH, Sen L, Cooke G, Dhanda R (2002). HIV-1 infection and AIDS dementia are influenced by a mutant MCP-1 allele linked to increased monocyte infiltration of tissues and MCP-1 levels.. Proc Natl Acad Sci USA.

[29] Tabara Y, Kohara K, Yamamoto Y, Igase M, Nakura J (2003). Polymorphism of the monocyte chemoattractant protein (MCP-1) gene is associated with the plasma level of MCP-1 but not with carotid intima-media thickness.. Hypertens Res.

[30] Mori H, Kaneko Y, Narita I, Goto S, Saito N (2005). Monocyte chemoattractant protein-1 A-2518G gene polymorphism and renal survival of Japanese patients with immunoglobulin A nephropathy.. Clin Exp Nephrol.

[31] Simeoni E, Hoffmann MM, Winkelmann BR, Ruiz J, Fleury S (2004). Association between the A-2518G polymorphism in the monocyte chemoattractant protein-1 gene and insulin resistance and Type 2 diabetes mellitus.. Diabetologia.

[32] Wright EK, Page SH, Barber SA, Clements JE (2008). Prep1/Pbx2 complexes regulate CCL2 expression through the -2578 guanine polymorphism.. Genes Immun.

[33] Ansari A, Talat N, Jamil B, Hasan Z, Razzaki T (2009). Cytokine gene polymorphisms across tuberculosis clinical spectrum in Pakistani patients.. PLoS ONE.

[34] Talat N, Shahid F, Dawood G, Hussain R (2009). Dynamic changes in biomarker profiles associated with clinical and subclinical tuberculosis in a high transmission setting: a four-year follow-up study.. Scand J Immunol.

[35] Carr MW, Roth SJ, Luther E, Rose SS, Springer TA (1994). Monocyte chemoattractant protein 1 acts as a T-lymphocyte chemoattractant.. Proc Natl Acad Sci U S A.

[36] Siveke JT, Hamann A (1998). T helper 1 and T helper 2 cells respond differentially to chemokines.. J Immunol.

[37] Mendez A, Hernandez-Pando R, Contreras S, Aguilar D, Rook GA (2011). CCL2, CCL18 and sIL-4R in renal, meningeal and pulmonary TB; a 2 year study of patients and contacts.. Tuberculosis (Edinb ).

[38] Tapaninen P, Korhonen A, Pusa L, Seppala I, Tuuminen T (2010). Effector memory T-cells dominate immune responses in tuberculosis treatment: antigen or bacteria persistence?. Int J Tuberc Lung Dis.

