# Supplementary figures and images for: CCL2/MCP-I Genotype-Phenotype Relationship in Latent Tuberculosis Infection
Source: PLoS One. 2011 Oct 4;6(10):e25803. doi: 10.1371/journal.pone.0025803 (PMC3186769; doi:10.1371/journal.pone.0025803)

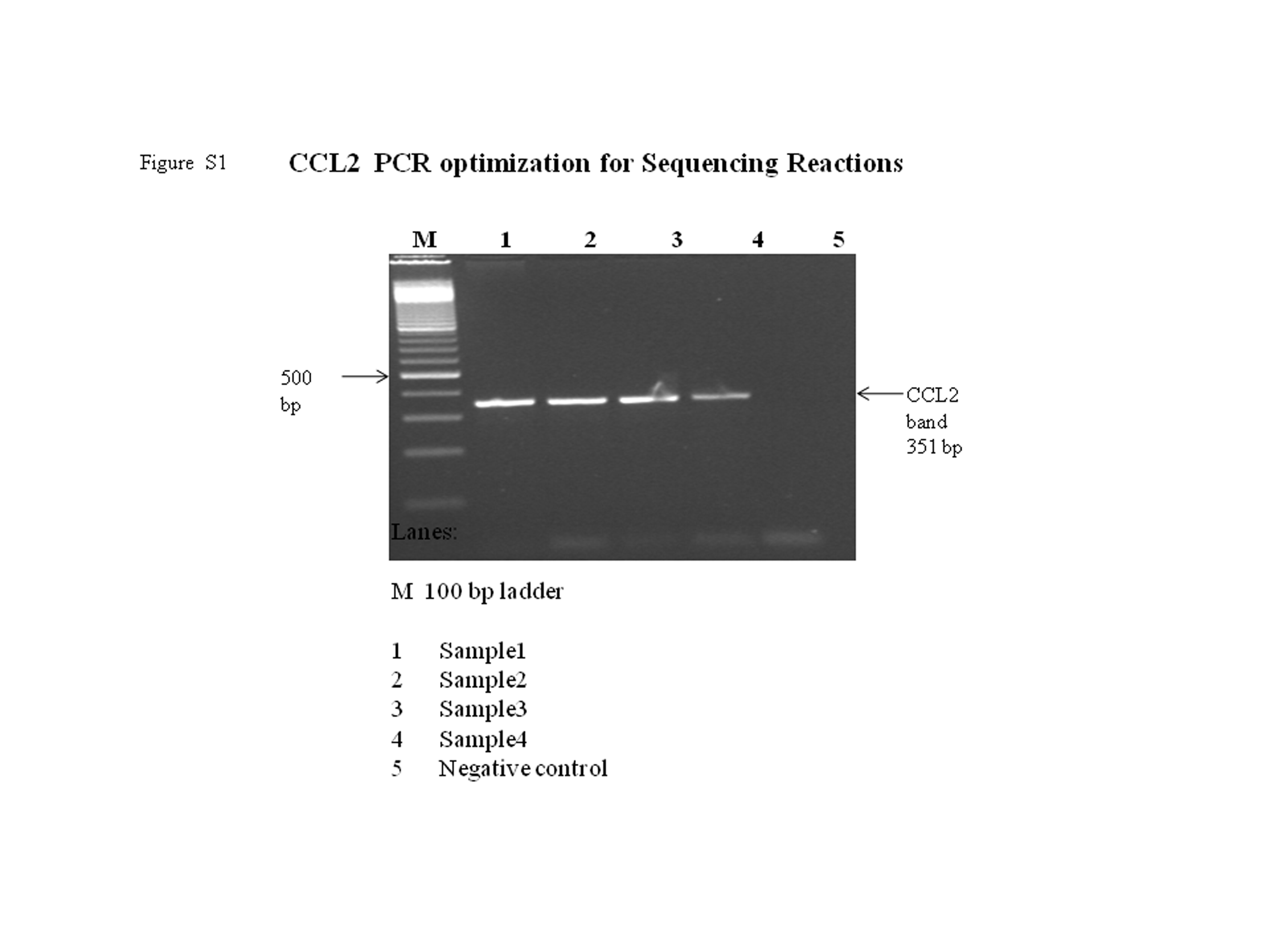

Supplement: Figure S1 — CCL2 PCR optimization for Sequencing Reactions. (TIF) [file pone.0025803.s001.tif]
