# Supplementary material for: CCL2/MCP-I Genotype-Phenotype Relationship in Latent Tuberculosis Infection
Source: PLoS One. 2011 Oct 4;6(10):e25803. doi: 10.1371/journal.pone.0025803 (PMC3186769; doi:10.1371/journal.pone.0025803)
Supplement: Table S1 — Frequency of CCL2 SNPS in reported in different populations. (DOCX) [file pone.0025803.s002.docx]

| **Table S1** |  |  |  |  |  |  | **Genotype %** | | | **Alleles %** | |  |  |
| --- | --- | --- | --- | --- | --- | --- | --- | --- | --- | --- | --- | --- | --- |
| **Author** | **Year** | **Ethnicity** | **Study groups** | **N=** | **CCL-2 snp -2518** | **HWE** | **GG** | **AG** | **AA** | **G** | **A** | **P-value** | **OR (95% CI)** |
| [1]et al | 2011 | Tunisian | TB | 223 | AA | No | 12 | 39 | 49 | 31.5 | 63.5 | p=0.01 (pc=0.03) | 0.6 (0.38–0.93) |
| Ben-Selma et al | 2011 | Tunisian | Controls (PPD?) | 150 |  | Yes | 5 | 33 | 62 | 21.5 | 64 |  |  |
|  |  |  |  |  |  |  |  |  |  |  |  |  |  |
| [2] et al | 2010 | Peruvians | PTB | 701 | GG | Yes | 50 | 39 | 11 | 69.5 | 30.5 | 0.036 | 1.43 (1.02–2.0) |
| **Ganachari et al** | **2010** | **Peruvians** | **Controls (PPD+)** | **796** |  | **Yes** | 41.2 | 46.5 | **12.3** | **64.45** | **35.55** | **p=0.0082; χ11** |  |
|  |  |  |  |  |  |  |  |  |  |  |  |  |  |
| Ganachari et al | 2010 | Mexicans | PTB | 193 |  | Yes | 48 | 40 | 12 | 136 | 64 |  |  |
| Ganachari et al | 2010 | Mexicans | Controls (PPD+) | 243 |  | **Yes** | 29 | 52 | 19 | 110 | 90 |  |  |
|  |  |  |  |  |  |  |  |  |  |  |  |  |  |
| [3] et al | 2009 | S. African | TB | 431 | NA | Yes | 6 | 33 | 61 | 22.5 | 77.5 | 0.24 | - |
| Moller et al | 2009 | S. African | Control (PPD?) | 482 |  | Yes | 8 | 36 | 56 | 26 | 74 |  |  |
|  |  |  |  |  |  |  |  |  |  |  |  |  |  |
| [4] et al | 2009 | Indian | TB | 155 | NA | Yes | 13.7 | 35.3 | 51 | 31.35 | 68.65 | NS |  |
| Alagarasu et al | 2009 | Indian | Control (PPD?) | 206 |  | Yes | 14.3 | 39.9 | 45.8 | 34.25 | 65.75 |  |  |
|  |  |  |  |  |  |  |  |  |  |  |  |  |  |
| [5] et al | 2009 | Chinese | TB | 200 | - | NA | 32 | - | - | - | - | <0.001 | - |
| Xu et al | 2009 | Chinese | Controls PPD? | 200 |  | NA | 13 | - | - | - | - |  |  |
|  |  |  |  |  |  |  |  |  |  |  |  |  |  |
| [6] et al | 2008 | Zambian | TB | 46 | AG | Yes | 2.2 | 50 | 47.8 | 27.2 | 72.8 | 0.01 | 2.8 (1.3-5.5) |
| Buijtels et al | 2008 | Zambian | Controls PPD+ | 119 | G | Yes | 3.5 | 26.1 | 70.4 | 16.55 | 83.4 | 0.04 |  |
|  |  |  |  |  |  |  |  |  |  |  |  |  |  |
| [7] et al | 2008 | Russian | PTB | 1440 | NA | Yes | 8 | 41 | 50 | 29 | 71 | 0.86 | 0.99 (0.89-1.11) |
| Thye et al | 2008 | Russian | Controls (PPD ?) | 1529 |  | Yes | 10 | 38 | 52 | 29 | 71 |  |  |
|  |  |  |  |  |  |  |  |  |  |  |  |  |  |
| Thye et al | 2008 | Ghana | PTB | 1964 | G (protective) | Yes | 3 | 28 | 69 | 17 | 83 | 0.0012 | 0.81 (0.73-0.91) |
| Thye et al | 2008 | Ghana | Controls PPD? | 2312 | -362 C | Yes | 4 | 32 | 64 | 20 | 80 |  | LD with -2518 |
|  |  |  |  |  |  |  |  |  |  |  |  |  |  |
| [8] et al | 2008 | Dene | Controls (PPD?) | 61 | - | Yes | 84 | 15 | 0.2 | 91.5 | 7.7 | - | - |
| Larcombe et al | 2008 | Cree | Controls (PPD?) | 42 | - | Yes | 64 | 31 | 5 | 79.5 | 20.5 | - | - |
| Larcombe et al | 2008 | Caucasians | Controls (PPD?) | 91 | - | Yes | 2 | 42 | 56 | 23 | 77 | - | - |
|  |  |  |  |  |  |  |  |  |  |  |  |  |  |
| [9]et al | 2007 | Chinese | TB | 412 | NA | Yes | 27.3 | 49.6 | 23 | 52.1 | 47.8 | 0.608 | - |
| Chu et al | 2007 | Chinese | Controls (PPD? blood donors) | 465 |  | Yes | 24.5 | 50.6 | 24.9 | 49.8 | 50.2 |  |  |
|  |  |  |  |  |  |  |  |  |  |  |  |  |  |
| [10] et al | 2007 | Holland | PTB peads | 26 | NA | Yes | 8 | 38 | 54 | 27 | 73 | 0.74 | - |
| Sterling et al | 2007 | Holland | Controls peads | 29 |  | Yes | 10 | 24 | 66 | 22 | 78 |  |  |
|  |  |  |  |  |  |  |  |  |  |  |  |  |  |
| [11] et al | 2005 | Mexican | PTB | 435 |  | Yes | 53 | 39 | 9 | 72 | 28 |  |  |
| Flores-Villanueva et al | 2005 | Mexican | Controls PPD+ | 334 | GG and G | Yes | 27 | 48 | 25 | 51 | 49 | 0.0003 | 2.3 (1.5-3.5)x=12.9 |
| Flores-Villanueva et al | 2005 | Mexican | Controls PPD- | 176 |  |  | 26 | 50 | 24 | 51 | 49 | 0.0026 | x=9.08 |
| Flores-Villanueva et al | 2005 | Korean | PTB | 129 | GG and G | Yes | 36 | 49 | 15 | 60 | 40 | 0.00063 | 6.9 (3.4-14.1) |
| Flores-Villanueva et al | 2005 | Korean | Controls (PPD?) | 162 |  | Yes | 13.6 | 45.7 | 40.7 | 36 | 64 |  |  |

NA= no association; NS= not significant; NA= no association; TST status was specified (PPD+/-) or not specified (PPD?)

Reference List

1. Ben-Selma W, Harizi H, Boukadida J (2011) . Mol Biol Rep

2. Ganachari M, Ruiz-Morales JA, Gomez de la Torre Pretell JC, Dinh J, Granados J, et al. (2010) 1. PLoS ONE 5: e8881.

3. Moller M, Nebel A, Valentonyte R, van Helden PD, Schreiber S, et al. (2009). Tuberculosis (Edinb ) 89: 189-194.

4. Alagarasu K, Selvaraj P, Swaminathan S, Raghavan S, Narendran G, et al. (2009). Indian J Med Res 130: 444-450.

5. Xu ZE, Xie YY, Chen JH, Xing LL, Zhang AH, et al. (2009). Zhonghua Er Ke Za Zhi 47: 200-203.

6. Buijtels PC, van de Sande WW, Parkinson S, Petit PL, van der Sande MA, et al. (2008). Int J Tuberc Lung Dis 12: 1485-1488.

7. Thye T, Nejentsev S, Intemann CD, Browne EN, Chinbuah MA, et al. (2009). Hum Mol Genet 18: 381-388.

8. Larcombe LA, Orr PH, Lodge AM, Brown JS, Dembinski IJ, et al. (2008) J Infect Dis 198: 1175-1179.

9. Chu SF, Tam CM, Wong HS, Kam KM, Lau YL, et al. (2007). Genes Immun 8: 475-479.

10. Sterling TR, Martire T, de Almeida AS, Ding L, Greenberg DE, et al. (2007) Pediatrics 120: e912-e921.

11. Flores-Villanueva PO, Ruiz-Morales JA, Song CH, Flores LM, Jo EK, et al. (2005). J Exp Med 202: 1649-1658.
